# Using consumer engagement strategies to improve healthcare safety for young people: An exploration of the relevance and suitability of current approaches

**DOI:** 10.1111/hex.13629

**Published:** 2022-10-28

**Authors:** Bronwyn Newman, Kathryn Joseph, Fiona E. J. McDonald, Reema Harrison, Pandora Patterson

**Affiliations:** ^1^ Centre for Health Systems and Safety Research (CHSSR), Australian Institute of Health Innovation Macquarie University Sydney New South Wales Australia; ^2^ School of Nursing and Midwifery, Centre for Quality and Patient Safety Research, Institute for Health Transformation Deakin University Burwood Victoria Australia; ^3^ Canteen Australia Newtown New South Wales Australia

**Keywords:** cancer care, consumer engagement, consumer participation, co‐production, patient safety, young people

## Abstract

**Background:**

Consumer engagement in health care is recognized as a critical strategy to minimize healthcare‐associated harms, however, little research has focussed on strategies to engage young people in healthcare safety. This study explores the suitability of commonly used engagement strategies, such as brochures, interactive bedside charts or apps, for young people (14–25 years) to improve their healthcare safety, with a focus on cancer care.

**Methods:**

Four qualitative online workshops were conducted (*N* = 19). Two workshops included young people who had experienced cancer (*n* = 6) and two workshops included staff who support young people who had experienced a diagnosis of cancer (*n* = 12). Evidence from a systematic review was used to develop case studies of existing strategies as a topic guide for the online workshops. Data were analysed using a framework method and template analysis approach.

**Results:**

Thematic analysis against the analytic framework led to the development of four principles for engagement with young people: empowerment, transparency, participatory culture and flexibility. The transition from being ‘looked after’ to young people being responsible for their own care was an integrative theme which intersected all elements of the framework.

**Conclusion:**

For service providers to engage with young people about safety issues in cancer services, the strategies employed need to be tailored to consider the transitional nature of being an adolescent or young adult. A systemic approach that incorporates flexible, tailored engagement strategies, education and empowerment of young people and healthcare providers is required to engage effectively with young people about safety in healthcare. These findings may have implications beyond cancer care.

**Patient or Public Contribution:**

Workshop content was developed with and by the CanEngage team, including the Consumer Advisory Group, who reviewed content and inform wider project priorities.

## 1 INTRODUCTION

Patient and carer engagement in health care is positioned by health agencies internationally as a critical strategy to improve the safety of patient care.^1^, ^2^ Patient safety can be described as the absence of preventable harm to the patient during the process of health care, and/or the reduction of risk or unnecessary harm associated with the care delivery process.^3^ Involving patients and carers in providing information, identifying concerns and detecting mistakes are some of the strategies that have been associated with reducing preventable harm.^4^ Engagement can also occur at the service and system level, by drawing upon patients and/or carers in establishing mechanisms to advance clinical governance to build safer systems and services of care.^5^, ^6^

Through using a broad range of methods, patient engagement occurs on a spectrum from consultation through to collaboration and partnership at the point of care through to policy making.^7^ There is a growing body of evidence about the use and effectiveness of patient engagement strategies to improve the safety of care for adults, with a recent review establishing the benefit of collaborative strategy development, user‐friendly design, proactivity and agency sponsorship.^8^, ^9^, ^10^ Less is known about the suitability and relevance of current patient engagement strategies with young people.^11^, ^12^

The suitability and relevance of patient engagement strategies for use with young people are increasingly pertinent in the context of a growing understanding of the distinctive needs of young people accessing health services. Considerations regarding engagement are particularly relevant for young people with chronic, complex or ongoing conditions such as cancer,^13^ in which treatments are often complex, and ongoing and involve decision‐making between various practitioners or services.^14^ Reliance on support networks for guidance about health‐related decisions and care has been recognized as integral, particularly in chronic conditions, and the level of support preferred by young people varies.^15^ For example, in cancer care, young people have identified family engagement in planning, decision making and day‐to‐day care as vital.^15^ There is also increasing awareness of the need for greater emphasis on improving the health literacy of young people experiencing cancer to improve outcomes.^16^ Varied definitions of the age range described as a young person, adolescent or young adults in research and health services also have implications for decision‐making and care processes, including engagement in discussions about safety issues.^17^ In this article, we have used the term ‘young people’ to describe adolescents and young adults aged 14–25 years.

We sought to address the evidence gap around the suitability and relevance of current patient engagement strategies with young people through an analysis with young people and their service providers in cancer services across Australia. Our enquiry addressed the following questions:

1. How relevant and suitable are current patient engagement strategies for young people?
2. What adaptations can be made to make current strategies more inclusive?

## 2 METHODS

This study received appropriate ethical approvals as per National Health and Medical Research Council guidelines.

### 2.1 Design

We used a qualitative research design using online workshops.

### 2.2 Setting

Young people using cancer services along with cancer service providers across Australia were accessed via Canteen, a national cancer support organization providing support services to young people aged 12–25 years who have had a cancer diagnosis or experienced cancer in their family.^18^

### 2.3 Sample

Eligible participants were either young people aged 14–25 years who had accessed cancer support services through Canteen Australia or providers of cancer support services for young people employed by Canteen. The minimum age of 14 for young people was set based on ethical requirements regarding the provision of consent to participate. Additional eligibility criteria were being able to communicate in English and having access to a computer or smartphone with internet connectivity for online participation. Participation of young people who identify as culturally diverse was encouraged, but not essential criteria.

Eligibility to participate was assessed by Canteen service providers and the research team. Based on recommendations for workshop research, we aimed to recruit between five and seven participants for each workshop.^19^ We opted to conduct two online workshops for staff and two for young people.

### 2.4 Recruitment

Canteen promoted the research project to young people consumers via Canteen's online platform and social media channels, including Facebook and Twitter. Canteen service providers also identified eligible young people. Young people indicated their interest in participating by contacting the Canteen research team directly using the contact details on the recruitment invitation and were then sent an email with the Participant Information Sheet and Consent Form.

Participants were allocated to an online workshop according to their availability. Young people consumers and health service providers were allocated to separate workshops to facilitate ease of discussion given the potential for young people and service provider participants to have had a prior relationship via Canteen. All young people eligible to access Canteen were invited to participate, however, only young people who had experienced a diagnosis of cancer chose to take part.

### 2.5 Topic guide

The topic guide was developed by the research team (B. N., K. J., R. H., F. E. J. M. and P. P.) and reflected the content of a wider project exploring patient engagement with regard to improving safety in cancer settings for ethnic minority consumers.^10^ Workshop content, including case studies, was reviewed by the Research Team and Consumer Advisory Group and adaptations were made. In addition, minor adaptations were made to frame the discussion around the concept of the suitability of engagement strategies for young people using cancer services in consultation with a partner agency with extensive experience in cancer support. The topic guide included introductions, a discussion of three case studies that exemplified the current modes and practices used to engage about safety in health care before concluding with the next steps for the research project and contact details for relevant support services. A summary has been included in Supporting Information: File 1 (Workshop topic guide).

### 2.6 Data collection procedure

Before the workshops, participants were emailed a one‐page document, which contained information about the topic schedule so that participants could be prepared for the discussion. Participants were invited to ask any questions and to identify if they had any accessibility requirements to facilitate their participation by contacting the research team. Following receipt of written consent, data collection occurred via online workshops using Zoom conferencing software, which were co‐facilitated by two members of the research team (B. N. and K. J.) and were 90 min duration. Participants were able to join using audio only or video conferencing according to their preference.

### 2.7 Data analysis

Codes were assigned with ‘W#’ indicating the workshop number, ‘P#’ the individual participant number and ‘S’ or ‘YP’ to specify service providers and young people. Data were analysed using the framework method according to Ritchie and Spencer in a four‐stage process.^20^ In Stage 1, we adapted an existing thematic framework that was developed as part of the wider research project by the research team (B. N., K. J., R. H., F. E. J. M. and P. P.) to address the experience of young people, leading to one additional framework element. An additional code ‘Impact of being a young person’ was added to the framework described in a related paper^10^ and provided as Supporting Information: File 2 (Coding framework). In Stage 2, the thematic framework codes were indexed across each transcript using Microsoft Word by one researcher (B. N.) and independently by a second researcher (K. J.) with discrepancies discussed between the two researchers and resolved. In Stage 3, the framework coding was reviewed and refined by the wider research team (R. H., F. E. J. M. and P. P.). In Stage 4, the research team reviewed and refined the themes until finalized. The concept of integrative themes has been adopted from the template analysis approach^21^ and used to explore data.

## 3 RESULTS

**Figure 1:**
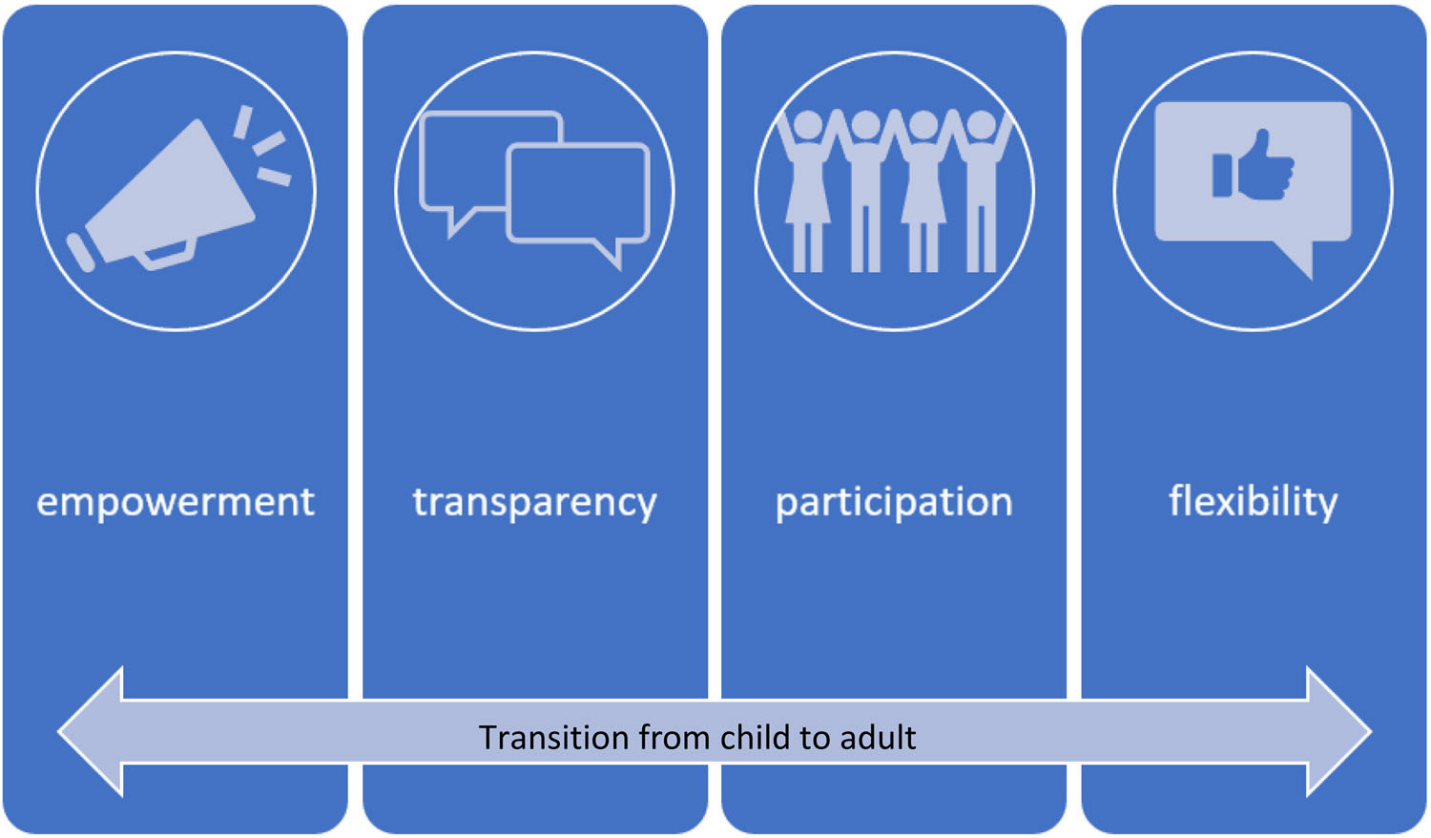
Principles for engagement with young people in health care

Four online workshops were conducted, two groups were for service providers who support young people experiencing cancer (*n* = 12) and two groups were attended by young people (*n* = 6). The young people who attended were from diverse cultural backgrounds and aged between 16 and 25. All young people participating had experienced a diagnosis of cancer and undertaken treatment in Australian health services. Thematic analysis against the analytic framework led to the development of four themes that are described as principles for engagement with young people: empowerment, transparency, participatory culture and flexibility (Figure 1).

### 3.1 Empowering young people to raise concerns

Workshop participants articulated that the health service environment often impacted the confidence of young people accessing care. Most participating young people and Staff participants were enthusiastic about engagement strategies to empower young people to raise concerns. Empowerment messaging is a key component of many engagement strategies and was central to one of the case studies explored in the workshops. The insight of one young person illustrates the impact of strategies that support young people accessing cancer services to speak up:

I think it's a very good thing because the more empowered you are, the more in control you feel and that's something you need to feel like you control something at least when everything is going to pieces around you. Being able—sort of—it's almost like being given permission to speak up even though maybe you shouldn't need permission but it's good. (young person #W4P1YP)

Participants highlighted both the importance of empowering young people to speak up, but also the complexity of this task. The quote below was representative of broader discussions:

Yeah, and I'm just thinking about people that perhaps don't have the same—they don't have a level of confidence in their communication to be able to go and say to a doctor or a nurse, hey, have you washed your hands. Or why are you doing that? You should be doing this. It just feels like it's a little bit—great idea but really just doesn't feel—to me, just doesn't feel like you would be empowering and useful for a—yeah, some people. Yeah. (staff member #W2P9S)

Staff members also explained the need to support young people to raise issues, both in describing the processes to raise a concern and providing relevant support, as illustrated in the following quotes:

The thing is that just because someone has an issue or difficulty speaking up or raising something, doesn't mean they don't want the empowerment. It just means they don't know how to go about it. (staff member #W1P1S)

I think just because a young person is being told not to be afraid to raise an issue, that doesn't mean they're not going to be afraid to raise an issue. (staff member #W1P4S)

Participants articulated numerous practical strategies to support empowerment, including opportunities to ask questions about how to raise concerns, and time to think through issues and discuss with supporters. Participants agreed that often the most significant impact on the empowerment of young people is their relationship with the person who is delivering the invitation to engage:

…bringing it back to that power imbalance of who is the health professional that goes through it with them and whether there is actual trust there in receiving the message that they're trying to give. Otherwise I think it sort of leads towards tokenistic empowerment. (staff member #W2P3S)

### 3.2 Transparent processes in health services

Several staff participants spoke about transparency and clarity in health system processes, seeing these principles as fostering trust between practitioners and young people. Participants said that strategies such as ensuring young people know they can be present for care‐related discussions and can access their notes if they choose to have the potential to increase young peoples' knowledge of health service processes and their likelihood of engagement. Participants spoke about the importance of young people knowing what is ‘normal’ to enable them to know when to raise a concern. This related to treatment issues, such as infection symptoms or side effects, as well as healthcare systems or processes, such as admission procedures or complaints processes. One staff member commented;

So like letting them know what their rights are, how things should operate, what they should be like and if they're not like that you also have the right to then speak up…. just knowing what you're rights are and what you can demand and what you can access I think is really good. (staff member #W2P5S)

At one workshop, young people discussed the importance of knowing who would see the information when a safety issue was raised and the steps to determine what would be done to remedy the issue. The relationship between healthcare staff, young people and parents was a central consideration in the discussion about trust and transparency in reporting and record keeping. One young person said: ‘I think as long as there's some sort of security, where if parents can ask questions that they don't want the kids to know and kids can ask questions they don't want the parents to know’ W3P4YP. Staff discussed the benefit of clear, transparent systems to increase the confidence of young people to raise issues and ask questions. One staff member said:

[Transparency] kind of removes that smoke and mirrors‐ness of if you didn't know what the process, what goes on behind closed doors, it can really help balance out that power and give that power back—in a way that if you did have safety concerns, you kind of know a little bit more about the process. (staff member #W1P2S)

### 3.3 Healthcare service culture to foster communication about safety

A consistent message from participants was the importance of a culture that normalises patient participation in discussions about safety issues. Many staff and young people discussed the challenge for young people to have the confidence to raise concerns, particularly in an adult hospital. Participants suggested that strategies to facilitate conversations between practitioners and young people about potential safety issues, without young people having to take the lead were valuable. Simple options such as a poster or brochure with a system to follow or words to use, for example, were proposed by young people. All workshops reinforced the importance of involving young people in the development of strategies and ensuring that images and strategies are inclusive and representative of young people. One staff member commented: ‘My thought is always, ask them…. in seeing what actually best suits that particular young person, or a group of young people. Because they're always going to know what they need more than any of us in this room are going to know what they need’ (staff member #W1P2S).

Young people also commented that consistent staffing with good rapport makes it easier to feel like there is a forum to raise concerns, especially if this contact can be across inpatient and outpatient stays. One young person described her experience;

My hospital had something similar where there was a nurse who's work number I got given…. Yeah so, I'd text her if I was having a bad day or bad symptoms or—and sometimes she'd tell me I need to go—she'd tell me to head into rapid assessment or do whatever [unclear]. Oh, in hospital she would pop in and come and check on me every day… (young person #W4P2YP)

### 3.4 Flexible approaches to patient engagement

More flexible approaches were suggested as ways to appeal directly to young people about care‐related decisions and enhance their awareness of opportunities to raise concerns about potential risk or safety issues. The principle of flexibility related to various elements including the timing of relevant information about services or diagnosis, as one young person said:

everyone's ready at different times. Like me I was pretty keen early on to see, you know whatever but I just not really sure how to ask and so I think making people aware—making people certain that there are things [information and support] you can access. Just come at your own pace. (young person #W4P2YP)

Another participant spoke about the importance of flexibility in the location of face‐to‐face engagement ‘I think allowing the young person to have some say where you discuss these things. Like if they're an inpatient. Just to give them a bit of choice’ (W2P2S). Flexibility is also related to offering standard or essential information resources in varied formats tailored to appeal to and meet the needs of young people including the physical design of brochures or posters. Participants suggested the use of images that represent young people, bright colours and resources that are ‘less medically’ (young person #W3P2YP).

Creating processes and communication strategies to accommodate the varying preferences of young people and their families in engagement was seen as essential, particularly in light of the changing nature of the relationships of support. The young people in workshop 3 discussed the impact of infection and the awkwardness of raising issues such as handwashing for infection control with visitors at the bedside, as well as with hospital staff. One participant expressed that simple pictorial information or a poster with clear messaging about infection control may have assisted them to raise issues about related behaviour more freely with adults and children who visited at the bedside. Other young people spoke about the benefit of having the option of several alternatives for direct contact with health practitioners—for example, the opportunity to ask in‐person or text questions to a familiar nurse as issues arose or contact a specialist with questions about treatment via email.

Flexibility and resources to enable health practitioners to communicate with parents directly when appropriate were also emphasized by participants. This was particularly significant for one young person who spoke about the challenge of translating complex information about her own chemotherapy treatment for her parent. The young person valued the support of her parent and found that having translated information provided by healthcare providers and discussing directly with her parent was a valuable approach. Communicating directly with her parent enabled her parent to understand the treatment, expectations and side effects to support her care away from the hospital setting. Other participants highlighted the need for flexibility to adapt expectations in line with culturally appropriate practices. This was particularly highlighted in relation to person‐centred planning and empowerment focussed strategies. One participant commented: ‘to me this is sort of saying, your role to be an empowered person is to ask questions and speak up whether it's comfortable for you or not. But that's just—yeah’ (staff member #W2P3S).

## 4 TRANSITION

Research participants said that for young people, inhabiting the transitional period between childhood and adult had a significant impact on engagement about health and more specifically, health safety. This phase of transition impacted the messaging of engagement as well as the mode (how it was delivered). One participant captured the nature of the transition period and the need to tailor engagement accordingly:

It's a much bigger issue. You can't change the fact that young people are often treated either as children or as adults and that they're not really seen as a separate cohort, where they very much are developmentally. But I think you just have to try and make it [engagement about safety] as focused towards them as you possibly can and then hope that that will work. Because yeah, I think they definitely still want to be empowered and given the option. (young person #W1P4YP)

The four principles outlined above were all impacted by the experience of transition for young people from being ‘looked after’ by parents or carers to being responsible for their own care, this was a theme which intersected all elements of the framework. We, therefore, considered ‘transition’ an intersectional or integrative theme.^21^ Within this integrative theme were two subthemes of ‘relationships of support’ and ‘navigating adult healthcare services’.

### 4.1 Relationships of support

Relationships with both family and peers were identified as significant influences and support for young people, and the level of influence was often shifting. This sentiment was conveyed directly in conversations about the value of formal peer support programmes, and also in comments about feeling more comfortable raising issues and asking questions if that is what peers accessing cancer care are comfortable doing. One young person spoke about the benefit of the opportunity to ‘meet other people, other young people like me’ (young person #W4P2). Overwhelmingly participants identified that family, particularly parents, were a central point of influence and support for most young people. The relationships between parents and young people with cancer were identified as a vital source of support for many, yet often had a changing, and at times complicated role. Several young people said that parents have a role to identify potential safety issues and the right to be informed about health care, yet this was not always straightforward. For example, participants identified that there were times when young people would prefer not to discuss issues with parents; ‘here are some things that you wouldn't really want to discuss with your parent, yeah’ (W3P3), or parents ‘may not want them [young person] to know information about their own health, which is super common’ (staff member #W1P1S).

All participant groups agreed that decisions about information sharing varied significantly between individuals and situations. One young person said:

it has to be your own decision to make and you can then go [over to] parents to help you make the right decision. But as many times I've heard, like there's no right or wrong too, most of these experiences. So, I reckon every person will have their own way to process information and to think what's the best option. (young person #W3P2YP)

The participants universally agreed that obtaining health or treatment information was important to inform young people what to expect in relation to activities such as health service processes or treatment plans. This knowledge was seen as vital to enable young people to know whether service access or treatment is progressing as expected and to identify potential safety events, however, access to such information was at times compromised. Several participants said that healthcare providers often address parents, one Staff member stated ‘I think most health professionals would speak to the parents first, if they're present…’ (staff member #W1P1S). Participants identified the impact of common patterns of interaction between young people, families and health practitioners on the opportunities for young people to gather information and raise issues. One Staff member articulated the impact of the transitional phase as a young person commenting:

….They [young person] want to be informed because often parents are protecting or hiding information, or they feel like they're hiding information from them. I think health professionals sometimes—although they [young person] also I guess are seen as an adult, they're also not really seen as a full adult. They're in between. (staff member #W1P1S)

Both staff workshops explored issues about the difficulties young people experienced negotiating discussions about health. One participant commented that young people often want more information than they are offered:

Generally, young people want to be empowered and have autonomy over their health and—because they're often—yeah, like their parents are taking over or someone else is handling it all and they're really wanting some control back. (staff member #W1P1S)

Family interactions were also noted to be difficult for some young people to navigate in informal exchanges, and several participants identified that these challenges are at times compounded by cultural factors. For example, one young person described the uneasiness of the responsibility to convey safety information related to hand washing and infection risk to their large extended family who visited often. Several participants raised that young people with parents who did not speak English had the additional responsibility of translating information for parents. Participants discussed the added burden and responsibility of ‘keeping parents in the loop’ when young people from Culturally and Linguistically Diverse (CALD) backgrounds were receiving treatment. In addition to the practicality of lingual barriers, participants also identified that for many young people from CALD backgrounds there may be cultural norms of respect, and reluctance to question authority which can impact their willingness to engage about safety. One young person commented: ‘I've got family who would rather die than feel like they are infringing on someone's [health practitioners] time’ (young person #W4P1YP).

### 4.2 Navigating adult healthcare services

Participants identified that the move from paediatric to adult health services significantly impacted engagement between many young people and health services, including safety‐related issues. Staff said that for agencies working across Australian state boundaries the differing age of consent across Australia makes clear expectations about rights and responsibilities difficult in formal consent or decision‐making processes. This inconsistency was seen to compound assumptions and expectations of healthcare providers and families about opportunities for young people to be involved in decision‐making and engagement, including engagement about safety. Participants identified that for many young people an adult hospital is a new, unfamiliar and overwhelming system to access, and navigation support was critical.

Participants at all workshops discussed that it is difficult for many young people to initiate conversations with adult health practitioners. Discussion at one workshop for young people centred around the importance of consistent relationships. One young person relayed her experience with a nurse who she was able to contact while in the hospital and when at home. This relationship provided valuable opportunities for the young person to clarify treatment, and side effects and raise issues of concern. Another young person discussed the use of humour within these familiar relationships to raise uncomfortable issues such as hygiene or handwashing practices.

Reflections of participating young people were mirrored in Staff workshops. Several Staff participants raised the issue of a ‘power imbalance’ in relationships between healthcare providers and young people that impacted young people's confidence to raise concerns, including about safety:

Because in a medical setting it's worth considering that there's that power imbalance between the doctor and young person…. whether or not they see it (staff member #W2P3S)

Staff participants at one workshop discussed the impact of authority and ‘power’ in health systems on young people's willingness to interact with healthcare providers. Staff experiences reflected that young people's attitudes often ranged from some who resisted being instructed about what to do, to others who were reluctant to challenge or question instruction and prone to acquiesce. One staff member discussed the increased impact on the confidence of young people using adult health services: ‘Yeah and I think—yeah, the power balance stuff is even more when you're a teenager in an adult hospital. Yeah’ (staff member #W1 P1S). Staff indicated that the adult hospital environment further impacted the willingness and confidence of young people to engage with healthcare providers, particularly about safety issues. One Staff member commented:

they're [Young people are] in this really weird area where they're like given—like put in their setting—an adult setting but then if your parent's present, you're not given that information. Or not in a youth friendly way. Yeah, so I think there's just lots of stuff around that. (staff member #W1P1S)

Participants were unanimous that the particular needs of young people require accommodation in adult hospital settings to facilitate engagement about safety issues or potential concerns.

## 5 DISCUSSION

The transitions in relationships of support with family members and peers, and changes from paediatric to adult health services commonly experienced by young people, can significantly compromise the effectiveness of current patient engagement strategies. Inhabiting the transitional period between childhood and adulthood has a significant impact on the engagement of young people in health care and more specifically, health safety. Our findings suggest that current patient engagement strategies implemented to enhance safety require adaptation to accommodate the needs of young people. Tailored approaches are required to support the transition from paediatric to adult health services, particularly for young people experiencing chronic conditions such as cancer.^22^, ^23^, ^24^, ^25^ Our research addresses a significant knowledge gap in understanding the requirements of strategies for engaging young people in their healthcare safety and provides the basis for creating and testing relevant tailored strategies for this population.

The benefits of consistent relationships between young people and health staff as conduits for communication have been further illuminated in our research, and their potential to impact the capacity of young people to raise questions or discuss safety issues requires further exploration. Tailored strategies to engage with young people at the point of direct care may benefit from a focus on facilitating sustained quality relationships between young people and service providers, this is particularly so for young people from diverse cultural backgrounds, or who have diverse learning or communication needs.^26^ Establishing collaborative, consistent relationships is particularly important for young people experiencing chronic conditions such as cancer.^27^, ^28^ Principles established in existing work could be useful to guide more inclusive strategies and approaches to engagement about safety.^25^, ^26^ The benefit of digital technologies to foster ongoing connection was identified in our study reinforcing this is an area for further investigation.^29^ The responsibility of service providers to develop relationships with young people to tailor communication in cancer care has been highlighted,^27^ as having the benefits of establishing a consistent point of contact within the healthcare service for young people to access between appointments through interventions such as cancer navigators.^24^

Collaborative research is required to increase understanding about the strategies that may be effective to facilitate engagement, and how young people can be equipped to raise safety issues. Improving health literacy to enable consumers to identify is a vital component in empowering consumers, including young people, to raise concerns about safety or questions about treatment.^16^, ^30^, ^31^ Empowerment has been recognized as a foundation for partnership^32^ and the use of empowerment tools has increased significantly within health care internationally over recent decades, for example, in relation to specific issues such as infection control,^33^, ^34^, ^35^ medication^36^ or in interactions in activities such as rounds or service planning.^37^, ^38^, ^39^ A recent review conducted by Halvorsen et al.^40^ concluded that to genuinely empower, or transfer power, and enhance the confidence of health service users to engage or raise issues is challenging. Genuine inclusive empowerment requires a nuanced, tailored approach, and this has been noted in opportunities to contribute to safety.^41^ Greater inclusion of diverse populations in the development and evaluation of engagement strategies, including the role of empowerment, is required.^8^, ^42^ The absence of evidence about young people in research about engagement and the impact of the transitions experienced during this stage, highlights the need for further research.^12^

Targeted strategies to engage young people about safety require consultation and partnership with young people in strategy inception, development and implementation, as the benefit of participatory development of engagement strategies and processes in health care is well recognized.^7^ Data from the present study indicated a need for greater engagement of young people in the inception and creation of engagement strategies about safety related to direct care, as well as health service processes and policy. These findings reflected a recent review which noted that young people with chronic conditions are increasingly encouraged to participate in conversations and decision‐making about their care and highlighted the limited evidence about engaging with young people about systemic or governance issues.^27^ Opportunities for young people to engage in safety at direct care and service systems or governance levels, are required across both paediatric and adult health services and need to be inclusive of diverse and minority populations.^8^, ^43^ Partnership and co‐production of resources and strategies requires organizational commitment, including policy support and staff education, to create an environment which supports recognition of consumer opinions.^12^, ^44^ There is a growing call for recognition of the needs of young people in service development and governance, and opportunities for leadership to foster young people to be heard.^13^, ^45^

## 6 STRENGTHS AND LIMITATIONS

This research was novel as it explored the views of a focused group of participants with niche experience. All material developed to guide the workshops, such as case studies and PowerPoint slides, were amended in consultation with consumer advisors and cancer support staff. The workshop approach with discussion around existing strategies provided a narrow focus for detailed discussion with small, distinctive participant groups. The study size and inclusion of Australian‐only experiences is reflective of the niche population group, yet could also be viewed as a limitation of our work. The recruitment of staff from a single organization and a small sample from the young person population are also limitations to the generalizability of our findings. Additionally, the variation in definitions and terminology of a young person within the literature from Australia and internationally makes it difficult to find and compare evidence.

## 7 CONCLUSION

The effectiveness of current patient engagement strategies is impacted by the transitions in relationships of support with family members and peers, and changes from paediatric to adult health services commonly experienced by young people. The absence of evidence about young people in research that explores engagement about safety, and the impact of the various transitions experienced during this stage of life, highlights the need for further research. Sustained, quality relationships between young people and service providers are required to facilitate engagement, particularly for young people experiencing chronic conditions. Tailored, collaboratively produced strategies which enable and encourage interaction between young people and health practitioners are needed to establish and maintain such relationships.

## AUTHOR CONTRIBUTIONS

Reema Harrison and the CanEngage project team conceived the study. Two researchers (Bronwyn Newman and Kathryn Joseph) conducted workshops and analysed the data. Reema Harrison, Pandora Patterson and Fiona E. J. McDonald assisted in the development of the coding framework and confirmed coding decisions. Bronwyn Newman completed writing the manuscript. Reema Harrison, Pandora Patterson, Fiona E. J. McDonald and Kathryn Joseph provided feedback during analysis and reporting and reviewed the manuscript.

## CONFLICT OF INTEREST

The authors declare no conflict of interest.

## ETHICS STATEMENT

This study received appropriate ethical approval from the University of New South Wales Ethics Committee (reference number: HC200486).

## Supporting information

Supporting information.

Supporting information.

## Data Availability

Data are available on request due to privacy/ethical restrictions.
