# Supplementary material for: Using consumer engagement strategies to improve healthcare safety for young people: An exploration of the relevance and suitability of current approaches
Source: Health Expect. 2022 Oct 28;25(6):3215–24. doi: 10.1111/hex.13629 (PMC9700142; doi:10.1111/hex.13629)
Supplement: Supplementary file 1 — Supporting information. [file HEX-25--s001.pdf]

TOPIC GUIDE

A summary of 3 case studies was provided to participants to prepare for workshop discussion.

The case studies focused on common strategies used to engage patients accessing direct care. They incorporated a reporting tool for patients and/or care partners to discuss safety concerns with staff in person, strategies with an empowerment focus and online opportunities for reporting and interaction.

Workshop Question Guide

Questions were adapted by facilitators to explore strategies described in the case studies.

1. What are your initial impressions of the engagement strategy?
2. To what degree do you think the engagement strategy will be appropriate for young people?

What are some of the considerations for consumers from diverse backgrounds?
What things might work well?
What things might not work well?
What adaptations might make the engagement strategy feasible or more appropriate for young people?

3. How feasible is the engagement strategy for use in a cancer care setting?

What, if anything, would be needed to make it work?
