# Supplementary material for: Using consumer engagement strategies to improve healthcare safety for young people: An exploration of the relevance and suitability of current approaches
Source: Health Expect. 2022 Oct 28;25(6):3215–24. doi: 10.1111/hex.13629 (PMC9700142; doi:10.1111/hex.13629)
Supplement: Supplementary file 2 — Supporting information. [file HEX-25--s002.pdf]

Coding Framework	
**1. Communication (Communication as language)**	Interaction between service providers and consumers related to consumer engagement strategies
**2. Mode of engagement strategy design**	The way that engagement strategies are designed and presented.
**3. Consumer involvement in planning engagement strategies**	The way that consumers are consulted in the planning of engagement strategies
**4. Sociocultural factors and influences**	Ideas, values, beliefs that influence acceptance and appropriateness of consumer engagement practices
**5. Implementation of engagement strategies**	The way the consumer engagement strategies are carried out/organised in the clinical environment
**6. Messaging and focus of engagement strategies**	The information and content contained within the engagement strategy
**7. Risks and impacts of engagement strategies**	The impact and potential consequences of the engagement strategy (potential safety issues)
**8. Impact of being a young person.**	Issues related to being young, these include issues related to family, service provision and relationships.
**9. Other.**	Any issue not captured by the codes above.
